# Deciphering arable farmers’ intentions: Attitudes, norms, perceived advantages, and the influential role of group discussions on insect frass adoption

**DOI:** 10.1080/27685241.2025.2501385

**Published:** 2025-05-12

**Authors:** Kirstin L. Foolen-Torgerson, Jaap Sok, Marcel Dicke, Alfons G. J. M. Oude Lansink

**Affiliations:** a Business Economics, Wageningen University & Research, Wageningen, The Netherlands; b Laboratory of Entomology, Wageningen University & Research, Wageningen, The Netherlands

**Keywords:** Circular economy, diffusion of innovations, OLS regression, biopesticide, decision-making process, willingness-to-consider

## Abstract

Circularity in agriculture regarding the recycling of by-products from one form of production for use as inputs in another has become an urgent initiative as resources become more scarce and valuable. One potential example of circular agriculture is recycling the by-products of insect production (frass) as a crop and soil health promoter. This research investigates the drivers of arable farmers’ intentions to trial insect frass as an input on their farms using the Theory of Planned Behaviour and the Innovation Decision Process. In addition, the influence of group discussion participation on the drivers of farmers’ intentions is investigated to identify potential opportunities to influence the uptake of frass. Two questionnaires at two time-points (t_1_ and t_2_) were distributed to forty-six Dutch arable farmers. Between these time-points, half of the farmers participated in group discussions where their first impressions of frass were shared amongst each other. The results from several regression models suggest that in t_1_, farmers’ attitudes, perceived (descriptive) social norms and perceived behavioural control drive their intentions to trial frass. By t_2_, for those not in group discussions, attitudes were the only significant predictors of their intentions. For those who participated in the group discussion, the descriptive norm had a larger association with intentions than for those who did not. The results of this research contribute to an informed discussion on how group discussions, alongside policy-driven approaches, can serve as a mechanism for shaping perceptions and beliefs and influence the adoption of agricultural innovations like frass.

## 1. Introduction

As an alternative to waste generation and accumulation, a circular economy recognizes and capitalizes on the value of waste. By recycling by-products as useful inputs, a circular economy aids in minimizing unnecessary use of limited resources (Geissdoerfer et al., 2020). Agriculture has been identified as an industry where resources are avoidably leaking throughout the supply chain. There are opportunities for the by-products of one form of production to be recycled as valuable inputs for another; as such, the movement towards circular agriculture has become urgent (Dagevos & de Lauwere, 2021).

One example of a circular relationship exists between insect producers and arable farmers. The by-products of insect production – the insect manure, shed exoskeletons and uneaten feed, collectively termed “frass” (Barragán-Fonseca et al., 2022) – can be repurposed as a crop and soil health promoter for use on arable farms. Frass is an important by-product of the production of insects for food and feed, both in terms of biomass and economical revenues, with equal biomass output for insects such as black soldier fly larvae and frass (Gligorescu et al., 2020; Leipertz et al., 2024). With the projected increase in the production of insects for food and feed, the availability of frass will expand as well (Van Huis, 2020). Several mechanisms are suggested to be responsible for frass’ health promotion capabilities. For example, the shed exoskeletons in the frass contain chitin. Though chitin is not directly useable for the plant, its presence can trigger, for instance, the expression of defence-related genes (Parada et al., 2018) and can stimulate beneficial microbes that populate next to the plant’s roots (Bai, 2015). The beneficial microbes can digest the chitin. While doing so, the microbes make the nutrients of the frass more accessible and digestible, which boosts the plants’ potential yield and gives it the opportunity to prioritize and allocate its resources for self-defence purposes (Pangesti et al., 2013). Also, the beneficial microbes produce compounds that impede the growth of pathogens and herbivores via direct and indirect methods. Direct methods include being pathogenic when in contact with plant pathogens or herbivores (Cawoy et al., 2011; Kupferschmied et al., 2013); indirect methods include inducing the plant’s systemic resistance by activating hormonal signal transduction throughout the plant (Pieterse et al., 2014). For a more in-depth explanation of the expected crop and soil health promoting mechanisms of frass, we refer to Barragán-Fonseca et al. (2022). With such properties, frass can be a valuable and recycled input for arable farms.

Frass represents a promising circular input in agriculture that is not yet widely available in the market. To successfully diffuse into the market, it must be deemed a feasible input by farmers. Previous research has found that when considering the adoption of sustainable practices, farmers considered, among others, the cost effectiveness of the product, the farm’s future trajectory, the farmers’ opinions regarding environmentally friendly practices (Defrancesco et al., 2008), farmers’ risk aversion and the relative riskiness of the innovation (Ghadim et al., 2005), and social factors (Michel-Guillou & Moser, 2006). For a more detailed overview of the behavioural factors that affect farmers’ adoption of sustainable practices, see Dessart et al. (2019). However, much of what is already known regarding the drivers affecting farmers’ adoption behaviour was derived from reflective research – research that investigates farmers’ decision-making processes after having adopted the innovation (Rogers, 2003). In the case of insect frass, the innovation is not widely available for use, and therefore, most farmers have not yet been faced with the consideration to use insect frass. The literature lacks insights from the perspective of farmers’ decision-making process prior to the adoption of the innovation – also known as willingness-to-consider research (Dessart et al., 2019; Ma et al., 2012). Such insights are crucial for broadening the understanding of farmers’ adoption of sustainable practices like the uptake of insect frass. The gap in the literature is addressed in this research by investigating farmers’ decision-making process regarding insect frass as a crop and soil health promoter prior to an adoption or rejection decision.

In addition, there is a lack of understanding of how farmers’ decision-making process can be externally influenced from the perspective of willingness-to-consider research. Previous studies have investigated, among others, the use of group discussions to provide social reinforcement when decisions are being made (Cialdini, 2001; Lewin, 1952). Marra et al. (2003) found that sharing information with others and social learning were critical for farmers when considering the adoption of an agricultural innovation. This research builds on the premise of the influence of group discussions by examining how group discussions can influence farmers’ decisions regarding the use of insect frass. Such insights can be useful for effectively encouraging the uptake of circular agricultural inputs.

The objective of this research is thus two-fold: (1) to determine what drives farmers’ intentions to trial insect frass as a crop and soil health promoter and (2) to determine how group discussions affect the drivers of farmers’ intentions to trial insect frass. We do so by conducting this research prior to frass’ diffusion into the market from a willingness-to-consider perspective. This research provides practical insights for parties such as insect producers and policy makers that may be interested in the successful diffusion of insect frass in the future.

## 2. Theory

Two theoretical lenses were used to investigate what drives farmers’ intentions to trial insect frass – the Theory of Planned Behaviour (TPB) (Ajzen, 1991, 2012) and the Innovation-Decision Process (IDP) (Rogers, 2003).

### 2.1. Theory of planned behaviour

The TPB, from social psychology, posits that attitudes, perceived social norms and perceived behavioural control predict one’s intention to perform a given behaviour. Each of these predictors is broken down further into two sub-constructs. Attitudes (one’s disposition in favour or against the behaviour) are measured by the positive or negative felt experiences (experiential attitudes) and consequences (instrumental attitudes) perceived as being associated with the behaviour. Social norms (the perceived social pressure associated with the behaviour) are measured by the perception that others are or are not performing the behaviour (descriptive norm) and by the perception of what ought to be done (injunctive norm). Perceived behavioural control (PBC; the extent of control over the behaviour’s execution and capability of executing the behaviour) is measured by perceived capacity (one’s belief in his/her own capabilities associated with executing the behaviour) and autonomy (one’s belief regarding the control over the behaviour’s execution) (Fishbein & Ajzen, 2010).

The TPB has been readily applied in agricultural research to determine what motivates farmers’ behaviour. For example, Hijbeek et al. (2018) investigated the drivers of Dutch farmers’ intentions to increase the organic matter contents of their soil. Brazilian farmers’ intentions were investigated regarding the diversification of their production (Senger et al., 2017) and the adoption of natural grassland for cattle grazing (Borges et al., 2014). Zeweld et al. (2017) investigated the drivers of farmers’ engagement in sustainable agricultural practices. For a critical review of TPB research conducted in agriculture, see Sok et al. (2021). In accordance with the TPB, we test the following hypotheses:

H1a: Attitudes positively correlate with farmers’ intentions to trial insect frass.

H1b: Perceived social norms positively correlate with farmers’ intentions to trial insect frass.

H1c: Perceived behavioural control positively correlates with farmers’ intentions to trial insect frass.

Research on social influence and conformity has shown that behaviours are often performed to impress others (Cialdini & Trost, 1998). Specifically, descriptive norms influence behaviour – if someone else is performing a behaviour, others may follow suit (Cialdini, 2001). Werner and Stanley (2011) found that indeed descriptive norms played a role in the context of sharing leftover toxic home and garden chemicals with friends (instead of discarding them). Considering the findings of the previous research, we test the following hypothesis:

H1d: Participating in group discussions influences the role of descriptive norms as predictors of farmers’ intentions to trial insect frass.

### 2.2. Innovation-decision process

The IDP stems from technology adoption and communication research and is part of the Diffusion of Innovations theory (Rogers, 2003). It is a five-stage process where first knowledge about the innovation is gained, then an impression of the innovation is formed, and an adoption or rejection decision is made. If one chooses to adopt the innovation, then it is implemented and the decision of adoption is confirmed (Rogers, 2003). Provided that insect frass is still in its research and development phase, we investigate the second stage (the persuasion stage) of the IDP.

In the persuasion stage, how an individual perceives an innovation’s attributes is especially important when formulating their impressions towards it. Rogers describes five attributes an individual perceives: relative advantages, compatibility, complexity, observability and trialability. As frass is not widely available, nor is its effectiveness being openly demonstrated on test farms (making it observable), we focus on three of the attributes. The attributes are defined as the extent to which the innovation is perceived to be better than comparable products (relative advantage), consistent with one’s existing values, needs and past experience (compatibility), and difficult to use or understand (complexity) (Rogers, 2003). To further develop an impression, individuals may perform a forward-thinking exercise where they imagine applying the innovation within their situation (Rogers, 2003). This mental exercise dictates the individual’s intentions to trial the innovation.

The IDP has a long history of being applied in agriculture to grasp farmers’ perceptions of various innovations and innovative practices. As a few examples, researchers have investigated farmers’ perceptions of hybrid corn (Ryan & Gross, 1943), precision agricultural technology (Aubert et al., 2012), conservation practices (Mascia & Mills, 2018), and ecological intensification (Kernecker et al., 2021). In accordance with the IDP, we test the following hypotheses:

H2a: Perceived relative advantage positively correlates with farmers’ intentions to trial insect frass.

H2b: Perceived compatibility positively correlates with farmers’ intentions to trial insect frass.

H2c: Perceived complexity negatively correlates with farmers’ intentions to trial insect frass.

Interpersonal channels play an important role in the persuasion stage. This is because individuals may have doubts or uncertainties regarding the innovation; therefore, social reinforcement is sought to ascertain that their impressions are similar to their peers’ (Rogers, 2003). For example, Rosen (2000) describes how EndNote’s successful diffusion could be attributed to its diffusion through interpersonal networks. In that respect, group discussions can play a role of creating an interpersonal network situation; additionally, group discussions provide a platform where participants can learn what the group’s impression towards a given behaviour is, thereby creating social reinforcement (Cialdini, 2001; Lewin, 1952). Considering the findings of the previous research, we test the following hypothesis:

H2d: Participating in group discussions influences the farmers’ perceptions of the relative advantage, compatibility and complexity that predict their intentions to trial insect frass.

## 3. Materials & methods

### 3.1. Research design

The research was conducted by first assembling two groups of farmers: those who participated in group discussions with farming peers (Group A) and those who did not (Group B). Farmers in Group A came exclusively from study groups. This made organizing group discussions more convenient. Farmers in Group B came from study groups and via snowball sampling.

To test H1a-c, a questionnaire (denoted as the t_1_ questionnaire) was used. Prior to distributing the questionnaire, farmers needed to have (at least) a basic understanding of what insect frass was. Therefore, an informational video about insect frass was first presented to the farmers. The video was produced based on the findings of Torgerson et al. (2021) and presented (1) what insect frass is, (2) how it promotes the health of crops and soil, and (3) how farmers should apply it. The video encompassed the three types of knowledge in accordance with the first stage (the knowledge stage) of the IDP (Rogers, 2003). Use the following link to view the informational video with English subtitles: https://youtu.be/s4Y4t7uQo0s.

All farmers completed the t_1_ questionnaire immediately after watching the video. Eighteen of the twenty-three farmers in Group A completed a hardcopy of the t_1_ questionnaire before splitting off into discussion groups. The remaining five farmers in Group A could not be met in person due to COVID-19 restrictions. Therefore, they completed the t_1_ questionnaire via a link and then participated in a group discussion via Microsoft Teams.

For those who participated in the group discussions (Group A), the farmers were first asked to clarify any questions they had about the content presented in the video. The discussion leaders were specifically instructed on the types of questions they could answer to avoid providing additional information and introducing information bias into the group discussions. The discussion continued with the leaders asking, “What do you think of insect frass? Can you explain your stance for or against using frass on your farms, and what first impressions led you to this conclusion?” The leaders were instructed to encourage feedback from all farmers, ensuring no single participant or small group dominated the conversation. During the discussion, farmers were prompted to note down up to three keywords explaining their consideration or rejection of using frass on sticky notes. If the conversation waned, discussion leaders were equipped to stimulate further discussion with questions like: “Do you think Dutch arable farmers would be interested in using frass? What are the advantages or disadvantages of using frass compared to the soil and crop protection products you currently use? Would integrating frass into your farming practices be easy or difficult? Does frass align with your current or desired farming methods, and why?”.

After the discussion, the leaders collected sticky notes from the farmers, organizing them on a large sheet of paper divided into three sections: advantages, disadvantages, and questions/uncertainties. The group was then invited to discern the overall tone or general impression of the group. They discussed whether the consensus was generally in favour of, against, or indifferent to using insect frass on their farms and summarized the general conclusions. Each group discussion lasted approximately 30 to 40 minutes.

For twenty-three farmers who did not participate in the group discussions (Group B), the t_1_ questionnaire was provided in an emailed link immediately after the video to seventeen of them; the time in which the link to the video and questionnaire was open and recorded to ensure that they had taken enough time to watch the video and fill in the questionnaire, all of which did. The remaining six farmers in Group B were approached during a farmers’ study group session. After having watched the informational video, they completed a hardcopy of the t_1_ questionnaire; they did not participate in group discussions.

To address H1d, a second questionnaire (denoted as the t_2_ questionnaire) was distributed. For all farmers, the t_2_ questionnaire was distributed in person on their farms (with the exception of five conducted online due to restrictions related to the COVID-19 pandemic) within sixteen days of having watched the informational video.

The t_1_ and t_2_ questionnaires both measured the TPB constructs using the same questions. However, only the t_2_ questionnaire additionally measured farmers’ perceptions on the relative advantage, complexity and compatibility of insect frass. This was done to reduce the total amount of time the farmers spent filling in questionnaires. As such, H2a-d were addressed using the data obtained from the t_2_ questionnaire. Demographics were also collected in the t_2_ questionnaire.

**Figure 1.**
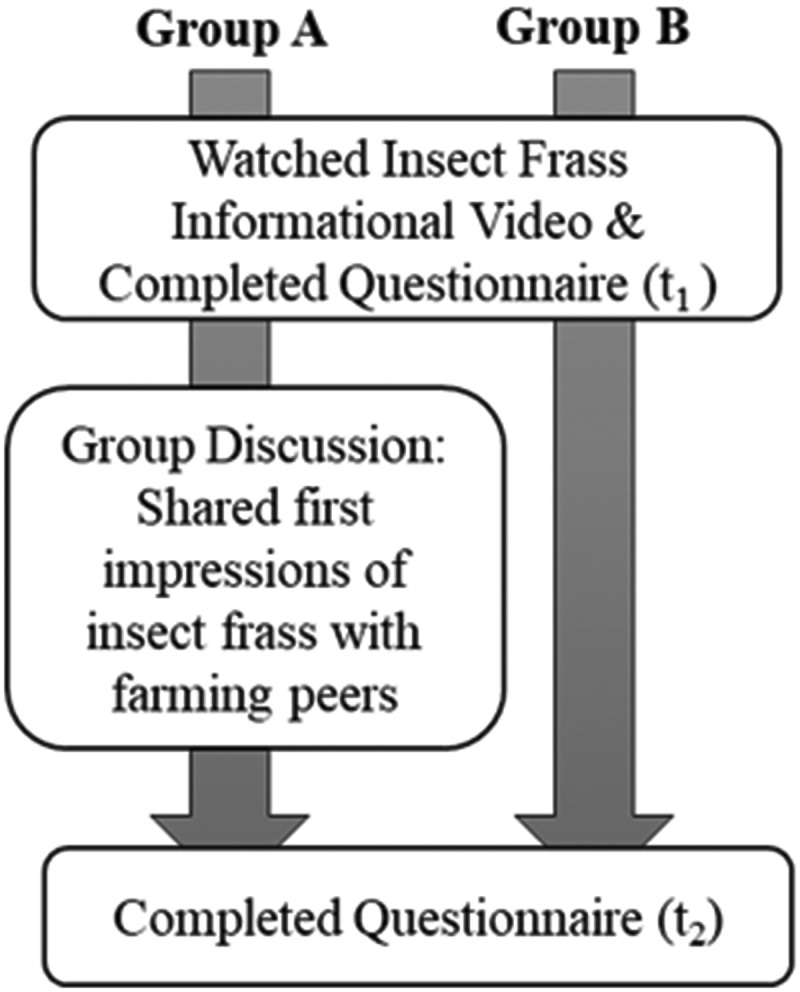
Research design.

Figure 1 presents a schematic overview of the research design. Table 1 presents an overview of the t_1_ and t_2_ questionnaires regarding the constructs, variables, statements in the questionnaire, scales and references for the questionnaire’s development.

**Table 1.** Description of variables for representing constructs in regression analysis.

TPB constructs - t_1_ & t_2_ questionnaires	Description of the statement^a^	Scale^b^
Attitude (instrumental)_1_	Using insect by-products [on parts of my arable cropping land within the next 5 years] is …	… unimportant – important
Attitude (instrumental)_2_		… disadvantageous – advantageous
Attitude (instrumental)_3_		… unnecessary – necessary
Attitude (experiential)_1_		… unsatisfying – satisfying
Social Norm (injunctive)_1_	Most people who are important to me would think that I should use insect by-products […].	
Social Norm (injunctive)_2_	The people who influence my decisions would think that I should use insect by-products […]	
Social Norm (descriptive)_1_	Most people like me would use insect by-products on a portion of their farm’s arable cropping land within the next 5 years.	
PBC (capacity)_1_	I am confident that I can use insect by-products […].	
PBC (capacity)_2_	If I really wanted to, I could use insect by-products […].	
PBC (autonomy)_1_	For me to use insect by-products […] is under my control.	
PBC (autonomy)_2_	The number of events outside my control which could prevent me from using insect by-products […] are …	… numerous – few
Intention_1_	I intend to use insect by-products […].	
Intention_2_	I plan to use insect by-products […].	
Intention_3_	I am willing to use insect by-products […].	
Perceptions constructs - t_2_ questionnaire	Description of the statement^c^	Scale^b^
Relative advantage_1_	[Compared to my currently used crop and soil health promoting products, using insect by-products] reduces my soil’s long-term susceptibility to pests and disease.	
Relative advantage_2_	[…]reduces the environmental impact of my activities.	
Relative advantage_3_	[…]improves my soil’s long-term quality (e.g. structure).	
Compatibility_1_	Using insect by-products is compatible with most aspects of my work (machinery, etc.).	
Compatibility_2_	Using insect by-products fits well with how my farm currently operates.	
Compatibility_3_	Using insect by-products fits well with the way I like to work.	
Complexity_1_	Insect by-products will be easy to use^d^	
Complexity_2_	Using insect by-products will be frustrating to learn.	
Complexity_3_	I clearly understand how to use insect by-products^d^	

PBC: Perceived Behavioural Control.

^a^Sources used for the TPB questionnaire development include: Aubert et al. (2012), Fishbein and Ajzen (2010), Taylor and Todd (1995), Vasquez et al. (2019) and Zeweld et al. (2017).

^b^The scale was disagree – agree, unless specified otherwise. All were 5-point Likert scales.

^c^Sources used for the perceptions (IDP – persuasion stage) questionnaire development include: Aubert et al. (2012), Moore and Benbasat (1991) and Taylor and Todd (1995).

^d^Variable was reversely coded for the statistical analysis.

### 3.2. Measurement and internal consistency of questionnaire design

The TPB items were formulated as direct measures of each construct, and as such, the TPB items represent reflective measures. Reflective measures must demonstrate internal consistency, unlike formative measures. As the perception items are formative measures, we did not assess the internal consistency of the indicators (Bollen & Lennox, 1991). However, we did check for multicollinearity in the model by inspecting Pearson and Spearman rank correlations and the Variance Inflation Factor (VIF). The correlation matrices did not reveal any concerning results, and the VIF results were all below 10.

The internal consistency of how well the TPB items collectively represented their assumed construct was checked within the t_1_ and t_2_ questionnaire output. Appendix A presents Tables A1-A4 that include the Kendall’s tau and Pearson correlation coefficients, means and standard errors for the TPB indicators for t_1_ and t_2_ respectively. The Kendall’s tau and Pearson’s correlations were compared to check for consistency, which were deemed robust to the various correlation specifications.

Cronbach’s alpha using Pearson correlation coefficients were calculated to check the internal consistency of the questions within their respective constructs based on the TPB (see Table 2). In addition, the (unstandardized) Cronbach’s alpha using covariances and the standardized Cronbach’s alpha using Kendall’s tau correlation coefficients were calculated to check that the results do not depend on the type of Cronbach’s alpha calculation conducted. The results were robust to the alternative Cronbach’s alpha specifications. Internal consistency is generally accepted at an alpha above 0.7 (Field, 2018).

**Table 2.** Standardized Cronbach’s alpha using Pearson correlation coefficients.

	Number of Indicators	Standardized Cronbach’s alpha (t_1_)	Standardized Cronbach’s alpha (t_2_)
Attitude	**4**	**0.79**	**0.73**
- Instrumental	3	0.74	0.71
- Experimental	1	NA	NA
Perceived Social Norm	**3**	**0.74**	**0.68**
- Injunctive	2	0.82	0.70
- Descriptive	1	NA	NA
Perceived Behavioural Control	**4**	**0.15**	**0.42**
- Perceived Capacity	2	0.61	0.68
- Perceived Autonomy	2	−1.10	0.27
Intention	**3**	**0.89**	**0.85**

The level of analysis was determined using the results from the standardized Cronbach’s alpha. Three levels of analysis were possible for the TPB items: construct-level, subconstruct-level, and the indicator-level (analysed per question). Attitudes were analysed at a construct-level, which consisted of the average of all four indicators measuring attitudes. The standardized Cronbach’s alpha was larger in t_1_ and t_2_ at the construct-level compared to the subconstruct-level. Perceived social norms were analysed at the subconstruct-level, which consisted of one indicator measuring descriptive norms and two indicators averaged to represent injunctive norms. In this way, we were able to address H1d, which tests the distinction between injunctive and descriptive norms. To represent perceived behavioural control, the standardized Cronbach’s alphas for the construct-level and subconstruct-level were unacceptable. Therefore, it was decided to analyse PBC on the indicator-level using only one of the items. Due external uncertainty around frass (e.g. legal allowance and availability of supply), the perceived autonomy indicators were not considered. The question, “I am confident that I can use insect frass on parts of my arable cropping land within the next 5 years” was selected because it provides an indication to farmers’ perceived capability to use insect frass. The items measuring intention resulted in the highest standardized Cronbach’s alphas. Intentions were therefore analysed at the construct-level where all three indicators measuring intentions were averaged.

### 3.3. Analysis of relationships

We conducted five of ordinary least squares (OLS) linear regressions and used robust standard errors to compute p-values. The regressions were conducted in R using “lm” function in the “stats” package (version 3.6.2).

In the first model, we addressed H1a-c by investigating which TPB constructs drive intentions in t_1_ (the dependent variable). The independent variables therefore included attitude, descriptive norms, injunctive norms and perceived behavioural control (capacity)_1_ from t_1_.

In the second and third models, we addressed H1d by investigating how group discussions influence the drivers of intention. To do so, we introduced the dummy variable “Group Discussion” to discriminate between farmers that participated in group discussion [1] and farmers that did not participate in group discussions [0]. The dependent variable was intentions in t_2_, and the independent variables included attitude, descriptive norms, injunctive norms and perceived behavioural control (capacity)_1_ and the product of these independent variables and the dummy variable.

In the fourth and fifth models, we addressed H2a-d by investigating perceptions from the IDP and the influence of group discussions on the perceptions. The dependent variable was intentions in t_2_, and all of the items corresponding to relative advantage, compatibility and complexity and the product of these variables and the dummy variable served as independent variables.

### 3.4. Sample demographics and data

Of the forty-six participating farmers, thirty-seven identified as conventional farmers, two as organic farmers, and seven as mixed (organic and conventional) farmers. Farms ranged in size (24–450 hectares) and percentage of land owned (0–100%). Farmers were almost exclusively male and ranged in years of experience (3–45 years), age (26–70 years old), and percentage of family income derived from the farm (10–100%) (see Table 3). Two-sample t-tests assuming equal variances were conducted, and no significant (at 5%) differences were found in the demographics of Table 3 between the two groups. The participants’ farms were located throughout the Netherlands.

**Table 3.** Demographics of farmers - groups A and B.

	Group A (group discussion)			Group B (no group discussion)		
	Min	Max	Average	Min	Max	Average
Land Owned (%)	0	100	61	0	100	67
Hectares of Arable Land	18	370	116	30	488	129
Years of Arable Farming Experience	3	45	28	4	45	27
Age	26	67	52	26	70	50
Family Income Derived from Farm (%)	10	100	77	35	100	83

Table 4 provides an overview of the means and standard deviations of all of the variables used in the OLS models. The table is split horizontally in two sections where the top section provides an overview of the data collected in t_1_, and the lower section provides an overview of the data collected in t_2_. A few farmers failed to answer all of the questions. The missing data points are also noted in the table.

**Table 4.** Descriptive overview of data.

	Group A (*n* = 23)		Group B (*n* = 23)		All (*n* = 46)	
Variable	Mean	St. Dev.	Mean	St. Dev.	Mean	St. Dev.
t_1_ questionnaire						
Attitude	3.19^a^	0.52^a^	3.38	0.70	3.29^b^	0.62^b^
Social Norm (descriptive)	2.96	0.93	2.52	1.28	2.74	1.12
Social Norm (injunctive)	2.69	0.85	2.48	1.06	2.58	0.96
Perceived Behavioural Control (Capacity)_1_	3.65	0.98	3.70	1.26	3.67	1.12
Intention	3.14	0.64	3.09	1.10	3.11	0.89
t_2_ questionnaire						
Attitude	3.13	0.51	3.14^a^	0.79^a^	3.13^b^	0.65^b^
Social Norm (descriptive)	3.00	0.83	2.70	1.15	2.83	1.00
Social Norm (injunctive)	2.78	0.74	2.59	1.22	2.69	1.00
Perceived Behavioural Control (Capacity)_1_	3.70	1.06	4.35	0.71	4.02	0.95
Intention	3.25	0.69	3.03	0.86	3.14	0.78
Relative Advantage_1_	3.35	0.71	3.46^a^	0.67^a^	3.40^b^	0.69^b^
Relative Advantage _2_	3.78	0.74	3.64^a^	0.95^a^	3.71^b^	0.84^b^
Relative Advantage _3_	3.57	0.59	3.46^a^	0.74^a^	3.51^b^	0.66^b^
Compatibility_1_	3.30	1.02	3.04	0.98	3.17	1.00
Compatibility _2_	3.65	0.78	3.26	0.96	3.46	0.89
Compatibility _3_	3.78	0.67	3.48	0.67	3.63	0.68
Complexity_1_	3.00	0.67	3.09	0.90	3.04	0.79
Complexity _2_	1.96	0.71	2.48	1.12	2.22	0.96
Complexity _3_	2.78	1.04	2.96	1.19	2.87	1.11

^a^22 observations (1 participant did not provide ratings).

^b^45 observations (1 participant did not provide ratings).

## 4. Results

### 4.1. TPB regression analysis

Table 5 presents three linear regression models. In Model A, attitudes, social norms (descriptive), social norms (injunctive) and perceived behavioural control (capacity)_1_ collected in t_1_ were implemented as predictors of intention. Model A shows that when all four predictors are regressed on intention, injunctive norms play an insignificant role.

**Table 5.** Linear models of TPB predictors of intentions.

	t_1_ analysis	t_2_ analysis	
Model Spec.	A	B	C
Exogenous constructs inserted	1–4	1–4	1–8
1. Attitude	0.76*** (0.15)	0.54* (0.21)	0.80** (0.27)
2. Social Norm (descriptive)	0.19 * (0.08)	0.18 (0.14)	0.00 (0.12)
3. Social Norm (injunctive)	0.13 (0.09)	0.08 (0.13)	−0.01 (0.17)
4. Perceived Behavioural Control (Capacity)_1_	0.19 ** (0.06)	0.10 (0.14)	0.05 (0.16)
5. Attitude x Group Discussion			−0.44 (0.37)
6. Social Norm (descriptive) x Group Discussion			0.48. (0.27)
7. Social Norm (injunctive) x Group Discussion			−0.02 (0.25)
8. Perceived Behavioural Control (Capacity)_1_ x Group Discussion			0.06 (0.17)
**Adjusted R**^**2**^	**0.67**	**0.43**	**0.47**

The level of significance is denoted with the following: *** for *p* < 0.001, ** for *p* < 0.01, * for *p* < 0.05 and . for *p* < 0.10. Robust standard errors (provided in parentheses below each coefficient) were used to compute the *p* values. The dummy variable was assigned 1 for Group A (those in the group discussion) and 0 for Group B (those not in the group discussion). Data from t_1_ was used in Models A. Data from t_2_ was used in Models B and C.

The lack of added value contributed by the injunctive norm is also demonstrated when each construct of Model A is broken down as a sole predictor of intention. In doing so, the injunctive social norms explain the least amount of variance of intentions (adjusted R^2^ is 0.12) compared to when perceived behavioural control (capacity)_1_, descriptive social norms or attitudes are independently regressed on intentions (adjusted R^2^ is 0.20, 0.41 and 0.54, respectively; see Appendix B). Model A was also run with interaction terms included (e.g. [construct] x Group Discussion) to check that there were no significant interaction effects. All of the interaction terms were insignificant (see results Appendix B).

The results from Model A suggest that without considering any additional affects from a group discussion intervention, attitudes, descriptive social norms and perceived behavioural control (capacity)_1_ are associated with intention. Therefore, H1a and H1c were not rejected. H1b was rejected in terms of injunctive norms but was not rejected in terms of descriptive norms.

Models B and C used data from t_2_. In t_2_, half of the farmers had participated in group discussions. Models B and C were constructed to compare how well the data explains the variance of intentions when not considering and considering (respectively) the additional effects of the group discussion. Model B therefore investigated the explanatory value of attitudes, social norms (descriptive), social norms (injunctive) and perceived behavioural control (capacity)_1_ with regards to intentions. In Model C, attitudes, social norms (descriptive), social norms (injunctive), perceived behavioural control (capacity)_1_ and the interaction of these four constructs with the Group Discussion dummy variable were used as the predictors of intention. Comparing the adjusted R^2^ of Model B with C, the variance of intention was better explained when including the interaction terms. In other words, accounting for the effect of the group discussion resulted in a better model fit than not accounting for the group discussion.

Furthermore, Model C shows that for those who did not participate in group discussions, attitudes were the only significant predictors of intentions; the role of social norms and perceived behavioural control (capacity)_1_ was insignificant. This result is also supported when investigating the role each construct with its subsequent interaction term has on explaining the variance of intention when used as sole predictors. Perceived behavioural control (capacity)_1_ and its subsequent interaction term explained the least amount of variance (R^2^ is 0.04). Descriptive norms, injunctive norms, attitudes and their subsequent interaction terms independently explained 21%, 26% and 39% of intention’s variance respectively (see Appendix C).

The group discussion resulted in an additional affect regarding the role of the descriptive norm. For those who did participate in the group discussion, their intentions to trial frass were more associated with the descriptive social norms (0.48; at *p* < 0.10) than for those not in the group discussion. Therefore, H1d, was not rejected.

### 4.2. Perceptions regression analysis

Table 6 presents Models D and E, which were based on farmers’ perceptions of frass’ relative advantages (compared to similar products), compatibility and complexity from t_2_. Model D evaluated how well the relative advantage, compatibility and complexity indicators alone explained the variance in intentions. Model E expanded on Model D by including the interaction terms (e.g. [item] x Group Discussion) to account for the group discussion. The results suggest that accounting for the group discussion produces a better fit model.

**Table 6.** Linear model of perception predictors of intentions (t_2_ analysis).

Model Spec.			E.1	E.2	E.3	E
**Relative advantage** *“Compared to my currently used crop and soil health promoting products, using insect by-products … ”*						
RA_1_		***“ …** reduces my soil’s long-term susceptibility to pests and disease”*	0.83 *** (0.13)			1.11 *** (0.21)
RA_2_		***“ …** reduces the environmental impact of my activities”*	0.25 * (0.10)			0.29 * (0.13)
RA_3_		***“ …** improves my soil’s long-term quality (e.g. structure)”*	−0.07 (0.12)			−0.36 (0.24)
RA _1_ x Group Discussion			−0.59 * (0.27)			−0.90 ** (0.27)
RA _2_ x Group Discussion			0.14 (0.16)			0.03 (0.20)
RA _3_ x Group Discussion			0.49 * (0.24)			0.70 * (0.29)
**Compatibility** *“Using insect by-products … ”*						
CB_1_	*“ … is compatible with most aspects of my work (machinery, etc.)”*			−0.01 (0.24)		−0.14 (0.19)
CB_2_	*“ … fits well with how my farm currently operates”*			0.34 (0.26)		0.23 (0.14)
CB_3_	*“ … fits well with the way I like to work”*			−0.06 (0.22)		−0.18 (0.17)
CB_1_ x Group Discussion				0.32 (0.36)		0.42 (0.25)
CB_2_ x Group Discussion				−1.01 ** (0.34)		−0.57 (0.36)
CB_3_ x Group Discussion				0.76 ** (0.26)		0.30 (0.50)
**Complexity**						
CX_1_		*“insect by-products will be easy to use”*			0.03 (0.16)	−0.08 (0.14)
CX_2_		*“using insect by-products will be frustrating to learn”*			0.04 (0.15)	−0.29 (0.21)
CX_3_		*“I clearly understand how to use insect by- products”*			−0.24. (0.13)	−0.06 (0.07)
CX_1_ x Group Discussion					−0.17 (0.25)	−0.10 (0.27)
CX _2_ x Group Discussion					−0.39. (0.23)	0.00 (0.34)
CX _3_ x Group Discussion					0.52 * (0.25)	0.19 (0.19)
Adjusted R^2^			**0.44**	**0.10**	**0.06**	**0.51**

The level of significance is denoted with the following: ***for p < 0.001, **for p < 0.01, and *for p < 0.05. Robust standard errors (provided in parentheses below each coefficient) were used to compute the *p* values. The dummy variable was assigned 1 for Group A (those in the group discussion) and 0 for Group B (those not in the group discussion).

Model E shows that for those who did not participate group discussions, two of the three measures of frass’ relative advantages (i.e. its ability to reduce the soil’s long-term susceptibility to pests and diseases [Relative Advantage_1_] and its ability to reduce the environmental impact of the farmers’ activities [Relative Advantage_2_]) were associated with intentions. Compatibility and complexity perceptions were not associated with intentions. A similar result was found when each construct (in Model E) with its subsequent interaction term was independently regressed on intention. Complexity indicators (and their subsequent interaction terms) explained the least amount of intention’s variance (R^2^ is 0.06). Relative advantage and compatibility indicators and their subsequent interaction terms independently explained 10% and 44% of the variance respectively (see Appendix D).

For those in the group discussion, two differences are found. First, the farmers’ perceptions of frass’ ability to improve the soil’s long-term quality (relative advantage_3_) were more associated with intentions for those in the group discussion. Second, the farmers’ perceptions of frass’ ability to reduce the soil’s long-term susceptibility to pests and diseases (relative advantage_1_) was less associated with intentions for those in the group discussion. This suggests that the group discussion influenced the relevance of some of the relative advantages of frass.

Overall, Model E suggests that for all the farmers, only frass’ relative advantages played a significant role in predicting intentions. Therefore, H2a was not rejected. Compatibility and complexity indicators played an insignificant role as predictors of intention; therefore, H2b and H2c were rejected. In addition, the relevance of various perceived relative advantages of frass differed for those in the group discussion than those not in the group discussion. H2d was therefore not rejected in terms of perceived relative advantages but was rejected in terms of perceived compatibility and complexity.

## 5. Discussion

### 5.1. Contributions and implications

This research set out to achieve two objectives. The first objective was to determine what drives farmers’ intentions to trial insect frass as a crop and soil health promoter. The results suggest that farmers initially base their intentions on multiple criteria, but only attitude towards frass dictate their intentions to trial it by t_2_. Therefore, attitudes that farmers develop based on the initial encounter with the information regarding frass are key. Additionally, for frass to successfully diffuse into the market, it is critical that farmers recognize the relative advantages frass has over comparable crop and soil health promoting products. As it is not expected that farmers decide to trial insect frass immediately after the first encounter, it is critical for frass’ successful diffusion that farmers have an initial positive attitude towards it and perceive its relative advantages.

The finding that initial attitudes critically influence farmers’ intentions to trial insect frass underscores the broader significance of first impressions in the adoption of agricultural innovations. This suggests that for effective diffusion of new sustainable practices or technologies in agriculture, the initial presentation and framing of information are crucial. Ensuring that farmers’ first encounters with these innovations are informative, positive, and address potential concerns can significantly enhance their willingness to adopt new practices, thereby advancing sustainable agriculture.

The second objective of this research was to determine how group discussions affected the drivers of farmers’ intentions. Using the TPB lens, the results suggest that participating in a group discussion significantly increased the importance of descriptive social norms. The importance of social influences on adoption decisions aligns with several other agricultural studies. For example, Borges and Oude Lansink (2016) found that farmers’ perceptions of the social pressures around improved natural grassland was the most important predictor for its adoption. Descriptive norms, more specifically, were found to play a role in the uptake of conservation tillage practices (D’Emden et al., 2008) and in the participation in agri-environmental schemes (Defrancesco et al., 2008). Similarly, membership in a farmer group was found by Meijer et al. (2015) to positively influence tree planting behaviour. In research experimenting with group discussions, Werner and Stanley (2011), who conducted group discussions to persuade individuals to share leftover toxic garden and home chemicals, found that persuasion was in part due to normative influences. In marketing research, Melnyk et al. (2011) found that the effect of the descriptive norm is increased when there is more cognitive deliberation. Granted, they also saw a subsequent decrease in the importance of the injunctive norm, which was not found in this research. Furthermore, participating in the group discussions may have provided the farmers with an opportunity to apply a heuristic shortcut where they could base their intentions more on what they perceived other farmers would do (Farrow et al., 2017).

Using the IDP lens, the results suggest that the group discussion influenced the relevance of various perceptions related to relative advantages for predicting intentions. The ideas shared between the farmers during the group discussion provide additional insights to the regression results. At least half of the groups discussed that frass, as a biological and natural product, could potentially reduce their use of other chemical products. This discussion point aligns with relative advantage_2_ measured using the statement, “compared to my currently used crop and soil health promoting products, using insect frass reduces the environmental impact of my activities”. However, the regression results suggest that discussing this topic did not influence its level of association with intention. This may be because after watching the informational video, this particular relative advantage was already clear and relevant.

Half of the groups also discussed to what extent frass was effective in the soil. This discussion point relates to relative advantage_1&3_ measured using the statements – “compared to my currently used crop and soil health promoting products, using insect frass …” “… reduces my soil’s long-term susceptibility to pests and disease” and “… improves my soil’s long-term quality”. As the group discussions were not audio recorded (only a written summary was recorded), further detail into these discussion topics is unknown. However, the results from the regression suggest that farmers may have been more convinced after the discussion of frass’ influence on the soil’s long-term improved quality than its reduced susceptibility to pests and disease.

In this research, we primarily focused on understanding farmers’ motivations for adopting sustainable crop protection practices based on reasoned opinions. Group discussions were used as an intervention to influence farmers’ willingness to trial frass. These discussions serve as a tool to initiate a change process by raising awareness about the benefits of adopting new practices, which, if convincing enough, can positively influence farmers’ intentions to adopt frass. However, a key first step before conducting the group discussions would be to ensure that there is enough evidence (also from test farms) that supports the acclaimed health promotion characteristics of frass. With the additional information at hand, a discussion can be facilitated in a way that farmers can share their initial impressions and express their concerns as a group, which can be addressed in the moment. In doing so, maybe the group’s impression of frass improves, influencing farmers’ individual attitude towards frass and their perception of what other farmers would do regarding the use of frass. Such an approach would be more effective than informing farmers individually and hoping their attitudes towards frass and their perceptions of frass’ relative advantages are positive. If positive impressions are shared amongst the group, farmers may believe that the others in the group would try using insect frass. In this way, the group discussions could potentially facilitate the uptake of frass.

The extrapolation of our group discussion-related findings to broader agricultural contexts offers valuable insights into the adoption of innovations in diverse farming practices. In high-tech agricultural settings such as precision farming or advanced horticulture, our research underscores the importance of facilitating dialogues among farmers. These discussions can extend beyond the mere technical advantages of new technologies, delving into the nuanced realms of social norms, attitudes, and peer influences. By creating forums for sharing experiences and perspectives, the adoption process can be enriched, emphasizing the less quantifiable yet crucial aspects of decision-making within these communities.

Similarly, in the context of livestock farming, particularly regarding the integration of sustainable practices like alternative feed sources, the role of group discussions assumes a pivotal position. Instead of solely relying on policy-driven approaches for implementing change, encouraging peer-to-peer interactions could lead to more authentic and voluntary adoption patterns. This strategy not only disseminates knowledge but also fosters a sense of communal understanding and shared goals, which are vital in the psychological landscape of decision-making among farmers.

In essence, the implications of our study transcend the specific case of insect frass adoption, offering a broader perspective on the dynamics of innovation acceptance in agriculture. It highlights the significance of understanding the subtler yet influential forces that shape farmers’ responses to new agricultural practices across various settings. Acknowledging the power of conversation and communal discourse is, therefore, imperative in devising effective strategies for promoting progressive and sustainable agricultural practices. However, it is also important to acknowledge that, to effectively increase the uptake of frass as a sustainable crop protection practice, additional and complementary interventions may be required to target behavioural factors beyond motivations driven by reasoned opinions (Finger et al., 2024; Sok et al., 2024).

### 5.2. Limitations

The data collection process of this research began prior to the COVID-19 pandemic; however, it was severely hindered as the lockdown in the Netherlands set in. Conducting group discussions in person was no longer possible. In an attempt to continue, we conducted one group discussion online. Though the discussion was still fruitful, the retention of participants was minimal. Therefore, we accepted the sample size that we managed to obtain.

The amount of time each farmer spent participating in our research was considerable. In addition, the farmers of course recognized the repetition in the TPB questions from t_1_ to t_2_, many of which commented on it while filling in the second questionnaire. Though it would have been interesting to test the perceptions hypotheses (H2a-d) in the same manner as the TPB hypotheses (H1a-d), the farmers would not have appreciated nine additional repeated questions, and it would have increased the time to complete the first questionnaire by more than 50%. Out of respect for the farmers’ time we chose to investigate the TPB lens in further detail and restrict the investigation of the perceptions lens to t_2_.

### 5.3. Future research

Several research directions can stem from this study. For instance, in this research, perceptions were not collected in t_1_. Therefore, a follow-up study could look into the perceptions that drive farmers’ initial intentions (in t_1_). As another example, this research investigated the drivers of farmers’ intentions at two time points in the persuasion stage. A questionnaire conducted at a third time point, specifically when an adoption (or rejection) decision is made can be used to determine if the drivers of intentions differ by the time the adoption stage is reached.

Another direction for future research can build on this research’s group discussion investigation. In this research, the group discussions were conducted in an open and unbiased way; the discussion moderators posed questions to the group that prompted the farmers to reflect on their own impressions. Future research could investigate whether nudging can be used to incentivize farmers similar to that conducted by Werner and Stanley (2011) to identify effective ways of promoting the adoption of green agricultural inputs amongst farmers. Notably, Dessart et al. (2019) discourages such approach with farmers because farmers are business oriented; nudging is intended for normal consumption persuasion.

Rogers (2003) discusses the importance of the change agent – one who attempts to influence the client’s innovation decision process towards (in the case of this research) trialling insect frass. Most often change agents are higher educated or possess technical knowledge regarding the innovation, and because of this, they are often not like the target group. This gap can cause communication challenges if not managed well. Therefore, in addition to introducing nudging into the group discussions, future research can also investigate which sorts of change agents such as young versus well-established university researchers, young versus well-established industry researchers or leaders of farmer study groups.

## 6. Conclusion

The objectives of this research were (1) to determine what drives farmers’ intentions to trial insect frass as a crop and soil health promoter and (2) to determine how group discussions affect the drivers of farmers’ intentions to trial insect frass. The results suggest that upon learning about insect frass, farmers’ attitudes, perceived descriptive norms and perceived behavioural control were associated with their intentions to trial insect frass. Within sixteen days after learning about frass, farmers completed a second questionnaire (in t_2_), which suggested that by t_2_, only farmers’ attitudes towards frass were associated with their intentions to trial it. Group discussions influenced the predictors of farmers’ intentions in two ways. Their beliefs that other farmers would trial insect frass were more important as a predictor of their intentions, and farmers’ perceptions of the relative advantages of frass differed between those who were and were not in group discussions.

This research underscores the wider importance of initial attitudes in the adoption of agricultural innovations. The findings highlight that the early framing and presentation of new technologies or practices, such as insect frass, can significantly influence farmers’ willingness to adopt them. It is essential for those introducing new agricultural methods to ensure that initial information is not only informative but also addresses potential concerns, creating a positive and receptive attitude from the outset.

The results of this research contribute to an informed discussion on how group discussions, alongside policy-driven approaches, can serve as a mechanism for shaping perceptions and beliefs and influence the adoption of agricultural innovations like frass. The impact of group discussions on shaping these attitudes emphasizes the value of community-based approaches in agricultural innovation adoption. Facilitating peer-to-peer dialogues and sharing experiences within farming communities can enhance the understanding and acceptance of new practices, making them more appealing and relatable. Such strategies, which foster communal learning and shared perspectives, are likely to be more effective in encouraging sustainable agricultural practices than traditional top-down approaches, aligning with the dynamic needs and preferences of modern farmers.
